# RoMIA: a framework for creating Robust Medical Imaging AI models for chest radiographs

**DOI:** 10.3389/fradi.2023.1274273

**Published:** 2024-01-08

**Authors:** Aditi Anand, Sarada Krithivasan, Kaushik Roy

**Affiliations:** School of Electrical and Computer Engineering, Purdue University, West Lafayette, IN, United States

**Keywords:** medical imaging, artificial intelligence, artificial neural networks, robustness, radiology, chest radiographs

## Abstract

Artificial Intelligence (AI) methods, particularly Deep Neural Networks (DNNs), have shown great promise in a range of medical imaging tasks. However, the susceptibility of DNNs to producing erroneous outputs under the presence of input noise and variations is of great concern and one of the largest challenges to their adoption in medical settings. Towards addressing this challenge, we explore the robustness of DNNs trained for chest radiograph classification under a range of perturbations reflective of clinical settings. We propose RoMIA, a framework for the creation of Robust Medical Imaging AI models. RoMIA adds three key steps to the model training and deployment flow: (i) Noise-added training, wherein a part of the training data is synthetically transformed to represent common noise sources, (ii) Fine-tuning with input mixing, in which the model is refined with inputs formed by mixing data from the original training set with a small number of images from a different source, and (iii) DCT-based denoising, which removes a fraction of high-frequency components of each image before applying the model to classify it. We applied RoMIA to create six different robust models for classifying chest radiographs using the CheXpert dataset. We evaluated the models on the CheXphoto dataset, which consists of naturally and synthetically perturbed images intended to evaluate robustness. Models produced by RoMIA show 3%–5% improvement in robust accuracy, which corresponds to an average reduction of 22.6% in misclassifications. These results suggest that RoMIA can be a useful step towards enabling the adoption of AI models in medical imaging applications.

## 1 Introduction

Artificial Intelligence is transforming the field of medicine in many ways, with applications spanning from drug discovery to genomics and, most prominently, radiology. Since AI has been particularly successful in computer vision, one of its most promising applications is to medical imaging. Deep neural networks (DNNs), which are composed of several layers of artificial neurons, have demonstrated great success in computer vision tasks. These networks, particularly convolutional neural networks (CNNs), have explored for various medical imaging tasks, including diagnosis of diabetic retinopathy (1, 2), breast cancer and malignant lymph nodes from histopathological images (3), and pulmonary and cardiological conditions from chest radiographs (4). The recent wave of promising research has led to significant interest in deploying these technologies in clinical settings. However, there are many hurdles that must be crossed before we can realize this potential.

Medical imaging models are first trained on a training dataset, and then tested in field trials before being deployed. One major challenge in this process arises from the differences between the data on which the models are trained and the data that they encounter after deployment (5). AI models are known to be very brittle to input noise and variations (6), even ones that are imperceptible to humans (7). There are several scenarios where medical imaging models encounter noise or variations that can impact the accuracy of their predictions (8). One popular use of medical imaging models is for telemedicine in areas that have a lack of trained physicians, where smartphones are used to take photos of scans, which are then sent through messaging apps, introducing distortion and compression artifacts (9). Additionally, using imaging equipment made by different manufacturers or using different settings on the imaging equipment can create variations in the resulting images (10, 11). AI models have also demonstrated significant performance variation across different patient populations (12). Any of these factors can result in a model making inaccurate predictions (8).

Recent work has demonstrated that variations and noise in the input can significantly reduce the accuracy of medical imaging AI models (8, 10). Although there has been a large body of work in the AI community on improving the robustness of these models under noise and adversarial perturbations, very few efforts have focused on the medical domain. There are various unique challenges posed by the domain of medical imaging that make it essential to address robustness specifically in this context (8). As described above, the nature of input noise and variations is primarily due to equipment differences, telemedicine, patient population; sources of variation seen in other settings (background objects, lighting, occlusion, etc.) are less relevant in medical settings (9, 10, 12). Furthermore, due to regulations and higher safeguards applied to medical data, adversarial attacks may be much less of a concern in this setting relative to other settings.

In this paper, we propose RoMIA, a framework to create more robust medical imaging models. RoMIA consists of three main steps: Noise-added Training, Fine-tuning with Input Mixing, and DCT-based denoising. In Noise-added Training, a fraction of the images in the training dataset are transformed by adding noise in order to make the trained model more robust (13). Specifically, we find that transformations such as glare matte, moire, and tilt result in models that perform best on photographs of radiographs. In Fine-tuning with Input Mixing, we fine-tune the trained model using a small amount of data from a different source in order to improve the model's robustness (14). Since only limited data from additional sources are likely to be available in practice, we use input mixing to avoid overfitting during this stage. Finally, in DCT-based denoising, we remove higher-frequency components in the input images before they are passed to the model for classification (15). This is motivated by our observation that perturbations encountered in medical imaging settings largely impact the high-frequency components of the images that are not essential for classification.

We evaluate the RoMIA framework using six popular CNNs trained on the CheXpert dataset, which contains 224,316 chest radiographs of 65,240 patients from Stanford Hospital (4). The created models diagnose Atelectasis, Cardiomegaly, Consolidation, Edema, and Pleural Effusion. For Fine-tuning with Input Mixing, we used 500 images from the ChestX-ray8 dataset from NIH (16). We evaluated the models using the CheXphoto dataset, which consists of 10,507 smartphone photos of chest radiographs from 3,000 patients (9). Our experiments indicate that a baseline model trained on the CheXpert dataset has an Area Under Receiving Operating Characteristic (AUROC) drop of 10%–14% when evaluated on the CheXphoto dataset. RoMIA creates models that improve AUROC by up to 5%, and reduces misclassifications by an average of 22.6%, underscoring its potential to create more robust medical imaging models.

### 1.1 Related work

Several research efforts have explored the use of CNNs for medical imaging. Building on these efforts, systems that support diagnosis are in various stages of deployment. These include systems for processing retinal scans (1, 2, 17), breast cancer detection (18), and skin cancer detection (19), among others. We focus our discussion on related efforts along two directions: those that explore CNN-based classification of chest radiographs and those that explore the robustness of medical imaging CNNs.

#### 1.1.1 Prior work on chest radiograph classification

Chest radiographs are among the most commonly requested radiological examinations since they are highly effective in detecting cardiothoracic and pulmonary abnormalities. Automation of abnormality detection in chest radiographs can help address the high workload of radiologists in large urban settings on the one hand, and the lack of experienced radiologists in less developed rural settings on the other. This need was only exacerbated during the COVID-19 pandemic when healthcare systems were overwhelmed and chest radiographs were commonly used as a first-line triage method. Motivated by this challenge, several efforts have developed DNN models for processing of chest radiographs (20–28). These works have proposed key ideas including the use of pre-training with natural images (20), multi-modal fusion of radiographs with clinical data (22), the use of transformer networks for such multi-modal fusion (24), manual design (27) or automated neural architecture search (25) to find a suitable DNN architecture for chest radiograph classification, bio-inspired training algorithms for small training sets (26) and the use of a focal loss function to address the significant class imbalance that is often present in chest radiograph datasets (28). These efforts have demonstrated high accuracies in various chest radiograph classification tasks, promoting interest in their use in clinical practice. Supporting the development of DNN models for chest radiographs has been the curation of public datasets (4, 9, 16, 29).

#### 1.1.2 Prior work on robustness of medical imaging AI models

It is well known that input variations, noise and adversarial perturbations can have a large negative impact on the accuracy of DNNs. For example, it has been shown that chest radiographs with added natural noise as well as the use of smartphone-captured photographs of radiographs caused significant degradation in accuracy (9). Another study found that DNN models trained on data from one hospital demonstrate considerably lower performance on data from a different hospital (10). Adversarial perturbations have also been shown to have a drastic impact on the accuracy of DNNs used in medical imaging (30, 31). These concerns, while broadly true of DNNs, are especially important for life-critical applications such as medical imaging. As a result, previous works have proposed and evaluated techniques to improve the robustness of medical imaging DNNs. The combination of large-scale supervised transfer learning with self-supervised learning was shown to improve the out-of-distribution generalization performance of medical imaging DNNs (32). The addition of Global Attention Noise during training (33), as well as adversarial training, where adversarial inputs are included in the training process (31), have been shown to improve the accuracy of medical imaging DNNs against adversarial attacks. Multi-task learning was used to address the specific challenges of prediction instability and explainability in the classification of smartphone photos of chest radiographs (21).

Our work makes the following contributions that go above and beyond the previous efforts. While noise-added training is a well-known technique to improve the robustness of neural networks (34) and has recently been applied to medical imaging specifically for adversarial robustness (31, 33), our work applies it to achieve robustness to natural sources of noise. Input mixing and DCT-based denoising have not been previously applied to the medical imaging domain to the best of our knowledge. Further, RoMIA is the first framework to combine these three techniques to improve robustness and to incorporate robustness improvement into all three key steps of the medical imaging AI pipeline (training, fine-tuning, and inference). Our results show that the combined use of all three techniques leads to substantially better accuracy than any of the techniques alone.

## 2 Materials and methods

In this section, we first describe the commonly used process for training medical imaging DNNs, and the challenges faced by such models due to input noise and variations. We then present the RoMIA framework to increase model robustness and the methodology used to evaluate it.

### 2.1 Pitfalls in conventional training methods

Typically, the creation of a medical imaging model starts with the collection of a large training dataset with training labels provided by physicians. In some cases, this may require years of data collection. For example, the CheXpert dataset of chest radiographs represents data collected over a period of 15 years (4). Next, a DNN is either trained from scratch or a model trained on a different computer vision dataset such as ImageNet (35) is transferred using the training data. The model may be evaluated on held-out or entirely different datasets, and then deployed. When deployed, the model may be applied to data that contains noise or variations. Frequently, this leads to significant degradation in model performance (8).

### 2.2 RoMIA framework

**Figure 1:**
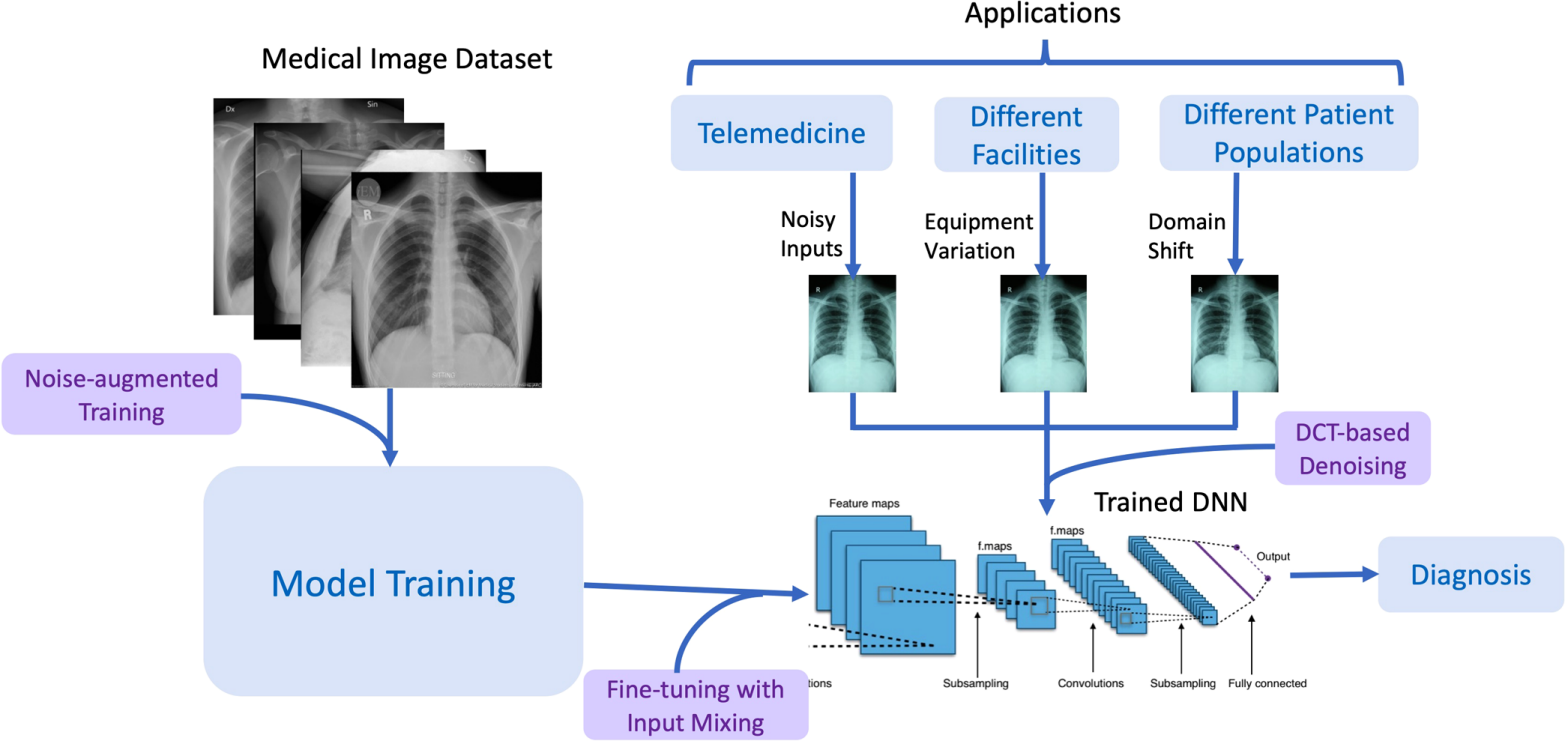
Overview of the RoMIA framework to create robust medical imaging AI models. The chest radiographs shown are from the CheXpert dataset (4).

Figure 1 describes the RoMIA framework to train more robust medical imaging models. We modify the standard model creation flow by adding three main components: Noise-added Training, Fine-tuning with Input Mixing, and DCT-based denoising.

#### 2.2.1 Noise-added training

In *Noise-added Training*, we introduce synthetic perturbations (noise) into the training data that mimic those observed in medical settings. We evaluated the following transformations:

• *Glare matte:* A filter designed to emulate the effect of glare observed when displaying the image on a matte screen.
• *Moire:* A filter designed to simulate the Moire effect, which produces repetitive interference patterns such as lines or stripes on the image due to limited resolution.
• *Tilt:* This transformation simulates a change in perspective that could result when a photograph of a medical image is taken using a device such as a smartphone (11).
• *Brightness* and *Contrast*: These transformations simulate changes to the settings in imaging equipment.
• *Blur:* This transformation simulates the loss in sharpness of the image due to motion of the patient during capture.

Among all evaluated transformations, we found that the first three were the most effective in creating more robust models. It bears mentioning that this result may be due to the fact that we evaluate robustness on the CheXphoto dataset. Hence, the transformations that introduce the most photographic noise may provide the best robustness. Notwithstanding this, the framework is extensible and additional transformations can be added to diversify the suite we have implemented.

We consider two strategies for applying noise to the training dataset: a specific percentage of the images in the dataset are injected with noise and either added (thereby expanding the dataset) or replace their original versions (thereby preserving the size of the dataset). We refer to these strategies as *augmentation* and *replacement*, respectively. All training hyperparameters (learning rate, batch size, optimizer, epochs, etc.) were kept unchanged.

#### 2.2.2 Fine-tuning with input mixing

In *Fine-tuning with Input Mixing*, we fine tune the model with a very small amount of data from a different source to improve the model's robustness. Since acquiring large amounts of additional training data may be challenging in practice, we limited ourselves to just 500 images, which correspond to around 0.22% of the original training set. While input mixing has been proposed in the literature as a data augmentation strategy, our contribution is the specific use of input mixing during the fine-tuning step and its evaluation in the context of medical imaging models. For our experiments, we draw these images at random from the ChestX-ray8 dataset from NIH (16). One challenge with using a very limited amount of data is that it could easily lead to overfitting. In order to prevent this, we use input mixing, a well-known technique where two images are combined into a composite input that contains information from both. Minimizing loss on mixed inputs has been shown to approximately correspond to maximizing robust accuracy (36). We mixed the additional data with images from the original training set for the fine- tuning phase. We considered three different mixing strategies that have been proposed in the literature. With CutMix (14), a randomly selected patch of one input image is placed into another. With MixUp, the pixels of two images are averaged in a weighted manner to construct a composite image. In both cases, the labels from the two images being mixed are also combined to derive the target label for the composite input (36, 37). In AugMix, images are mixed with augmented versions of themselves, so the label does not change (15). We mix the 500 images from ChestX-ray8 with 1,000 randomly selected images from the CheXpert training set and fine-tune the model for 3 epochs with these mixed inputs. All other hyperparameters such as the learning rate and optimizer were the same as those used in the training stage.

#### 2.2.3 DCT-based denoising

**Figure 2:**
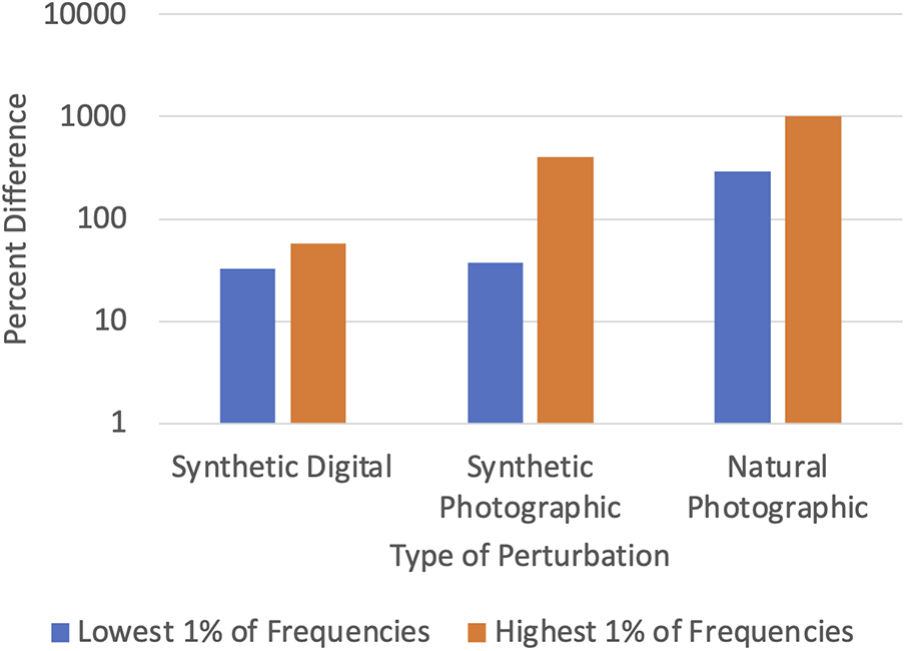
Difference between clean (CheXpert) and noisy (CheXphoto) images in high and low frequencies.

DCT-based denoising is based on the insight that most sources of noise disproportionately affect the high-frequency components of an image (38). This is shown in Figure 2, which plots the percent difference in the top and bottom 1% of frequencies of the original and noisy images from the CheXpert (4) and CheXphoto (9) datasets, where the noisy images were produced using synthetic digital perturbations, synthetic photographic perturbations, and photos taken of the images with a smartphone camera. During inference, we add a preprocessing stage to the model which uses DCT (discrete cosine transform) to transform the image into the frequency domain, then removes a set percentage of high-frequency components, and finally computes the inverse DCT (15, 39). The percentage of high-frequency components to be removed from an image (denoted by *η*) is determined through an experiment where a small fraction of the training set (CheXpert, in our experiments) is subject to DCT-based denoising for different values of *η*. For each model, the largest value of *η* (which corresponds to the most aggressive denoising) that keeps the AUROC to within 0.005 of the original accuracy (where *η* = 0) is chosen. Optimizing the hyperparameter *η* ensures that the frequencies removed do not significantly interfere with the features used by the model for classification.

To summarize, the proposed flow to create robust medical imaging models consists of transferring a model trained on ImageNet to the target medical imaging dataset using noise-added learning, then fine-tuning the resulting model with input mixing, then finally adding a DCT-based denoiser to the model before deployment.

### 2.3 Experimental setup

**Figure 3:**
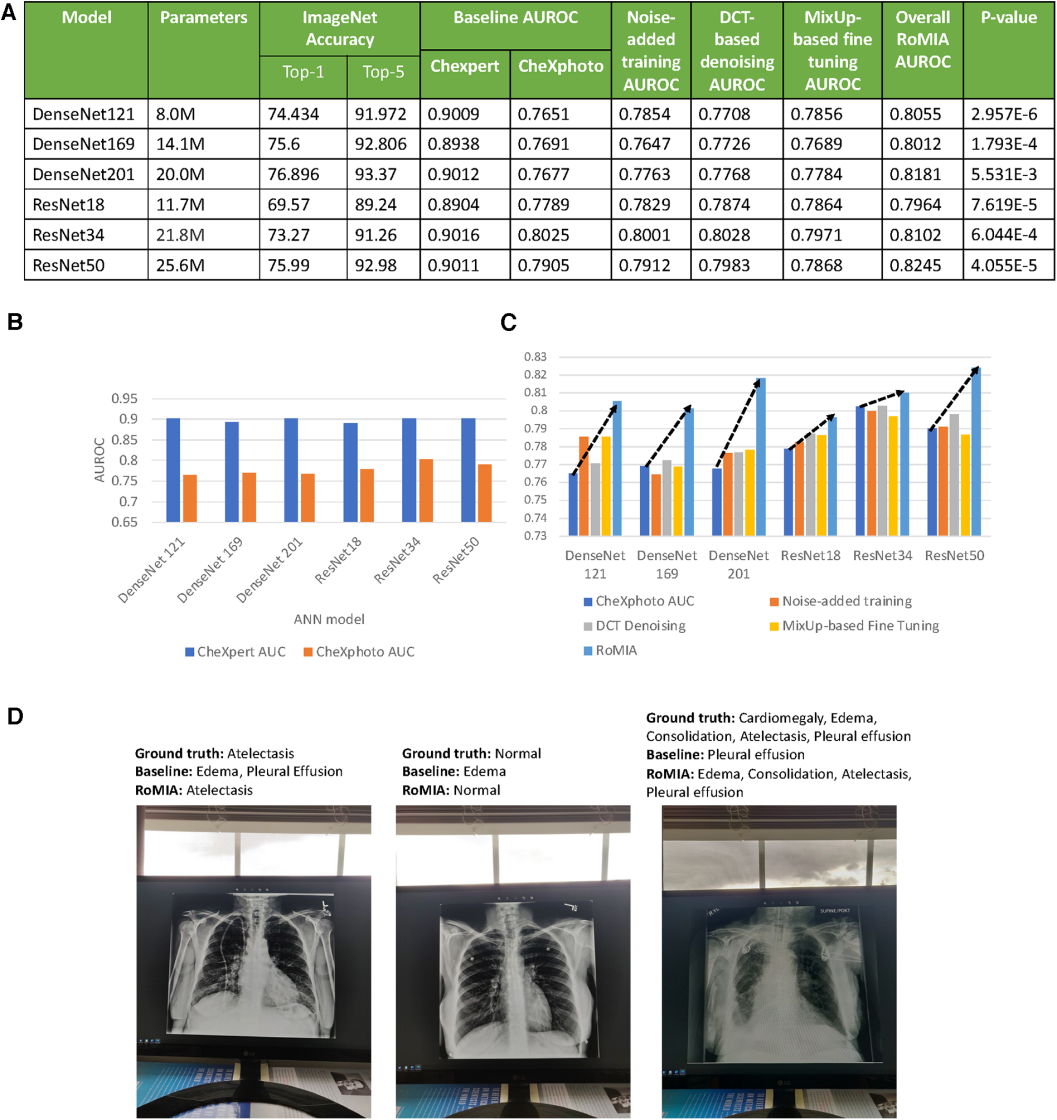
(**A**) Characteristics of the baseline models used in the experiments and accuracy values (**B**) AUROC of baseline models on CheXpert and CheXphoto, (**C**) AUROC improvement from RoMIA and each of its constituent techniques, and (**D**) example inputs misclassified by the baseline model but correctly classified by RoMIA model.

We implemented the RoMIA framework using the PyTorch (40), TensorFlow (41), libAUC (42), and OpenCV (43) libraries. We applied the framework to create models for classification of chest radiographs. The base models were selected from popular image classification DNNs trained on the ImageNet (35) dataset (see Figure 3A). Note that all the networks are Convolutional Neural Networks (CNNs), since these are the most popular type of DNN used for image classification tasks. We specifically created a model to detect Atelectasis, Cardiomegaly, Consolidation, Edema, and Pleural Effusion. Accordingly, the final fully connected layer of each base model was removed and replaced with a layer with five outputs. These models were then transferred using the CheXpert (4) dataset, which contains 224,316 chest radiographs of 65,240 patients from Stanford Hospital. For the fine-tuning step, we randomly selected 500 images from NIH's ChestX-ray8 (16) dataset. The learning rate used for both the transfer and fine-tuning steps was 0.0001, number of epochs was 3 with a batch size of 32, and weight decay was 10^−5^. The Adam optimizer and cross-entropy loss were used. For the MixUp (36) strategy, we use a beta distribution to select values between 0.4 and 0.6 to determine *λ*, the image mixing ratio. We evaluated the models on the CheXphoto (9) dataset, which consists of 10,507 natural photos and synthetic transformations of chest radiographs from 3,000 patients. Since our noise-added training step uses transformations similar to those in CheXphoto, we only perform our evaluations on the natural photographs. We repeated each of our experiments five times with different random seeds.

## 3 Results

In this section, we present results from evaluation of models created using the RoMIA framework. We first present the difference in AUROC of the baseline models when evaluated on a subset of CheXpert and CheXphoto images. Next, we present the performance of models trained using RoMIA and compare them to the baseline models. Subsequently, we perform an ablation study to investigate the contribution of each of the three components (Noise-added learning, Fine-tuning with input mixing, DCT-based denoising) to the overall improvement in robustness. We then explore different dataset transformation techniques for the Noise-added Training step and evaluate their impact on the model performance. We also compare the performance between different strategies for input mixing in the fine-tuning step. Finally, we explore the determination of the parameter *η* which controls the percent of high-frequency components removed from the input during DCT-based Denoising.

### 3.1 Robustness of baseline models

A key motivation for this work is that baseline models trained on a certain dataset perform significantly worse on similar datasets with added noise. To demonstrate this in the context of CheXpert and CheXphoto, we study the differences in AUROC of a baseline model trained on CheXpert and then applied to both CheXpert and CheXphoto data. Figure 3B presents the AUROC scores for the baseline models on the CheXpert and CheXphoto data. The figure shows a degradation of 10%–14% in AUROC across all six models, underscoring the need to create more robust models in the context of medical imaging.

### 3.2 Overall improvements from RoMIA and ablation study

The RoMIA framework consists of three techniques to improve robustness, so we conduct an ablation study to evaluate each component. Figure 3C shows the baseline accuracy, the results of the ablation study (applying each of the three techniques in RoMIA individually), and the resulting AUROC score when all three techniques are combined in RoMIA. To capture the benefits of the proposed framework, we first look solely at the CheXphoto AUROC values for the baseline and RoMIA models. We observe around 3%–5% improvement in AUROC, which corresponds to an average reduction in misclassifications by 22.6%, suggesting that the proposed framework is capable of creating substantially more robust models. We also observe a larger improvement in robustness on deeper models, such as ResNet50 and DenseNet201. We hypothesize that this is because deeper models can better learn the more diverse training data which they are presented in the RoMIA framework. In order to evaluate the statistical validity of the results, we repeated the training runs for the baseline and RoMIA models with 10 additional random seeds. We performed a one-tailed paired *t*-test and concluded that the improvements were statistically significant with *p* < 0.01. Figure 3D presents examples of inputs that are misclassified by the baseline model but correctly classified by RoMIA.

Figure 3C also presents the results of our ablation study to evaluate each of the three components in the proposed framework. We do this by evaluating the CheXphoto AUROC when each technique is applied individually. We observe that overall, each technique has a positive impact on robustness. The combination of three techniques used in RoMIA boosts AUROC by up to 5%. We evaluate each technique in more detail in subsequent sub-sections.

### 3.3 Contributions from noise-added training

**Figure 4:**
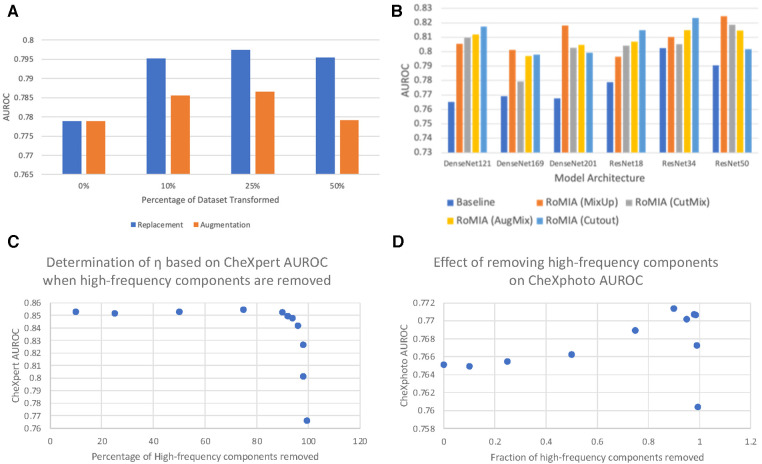
(**A**) Comparing dataset replacement vs. augmentation during noise-added training (ResNet18), (**B**) comparing different input mixing strategies in RoMIA for various networks, and (**C**) determination of frequency cutoff threshold (*η*) and (**D**) impact of *η* on CheXphoto AUROC.

Figure 4A explores the impact of various dataset transformation techniques used in noise-added learning. Specifically, we transformed 10%, 25%, and 50% of the training samples in the CheXpert dataset and either added them to the dataset (augmentation) or replaced the original samples with them (replacement). We observe that the 25% replacement strategy worked best across all networks. We note that this strategy does not impact training time, as the only overhead incurred is a one-time transformation (noise addition) to the inputs, which is insignificant.

### 3.4 Effect of fine-tuning with input mixing

Several approaches to input mixing have been proposed in the literature, primarily as methods for data augmentation that lead to better generalization of machine learning models. To evaluate the impact of the mixing strategy in the fine-tuning step of RoMIA, we consider CutMix (14) and MixUp (36), the two most widely used strategies, in addition to AugMix (15) and Cutout (44). To determine which strategy yields higher improvement in robustness, we compare in Figure 4B the AUROC boosts on the CheXphoto dataset when each strategy is applied. We observe that, while all mixing strategies yield improvements over the baseline, MixUp provides the best results overall, followed by Cutout and AugMix. This motivated our decision to use MixUp in the final RoMIA framework.

### 3.5 Selection of *η* in DCT-based denoising

A key feature of our framework is the DCT-based Denoising step, which removes high-frequency noise from the inputs. We use the parameter *η* to denote the percentage of high-frequency components removed from each image. In Figure 4C, D, we consider the impact of the choice of the parameter *η* by showing how different *η* values affect CheXpert and CheXphoto AUROC. Due to the nature of x-ray radiographs, we find that removing a large fraction of the high frequencies does not have a detrimental impact on performance for either dataset and in fact improves accuracy on the noisy (CheXphoto) data. We determine *η* as the largest value that results in a less than 0.5% decrease in accuracy on the clean (CheXpert) dataset (Figure 4C). We observe that this value of *η* improves performance on the CheXphoto dataset (Figure 4D). This result underscores the efficacy of DCT-based denoising.

## 4 Discussion

The success of AI in recent years has led to significant interest in applying it to the medical field. In particular, since DNNs have been very successful in image processing applications, they are frequently being applied to medical imaging tasks. One of the challenges that must be addressed when applying AI to any critical application, and certainly to medical imaging, is their robustness under conditions encountered in the real world. Previous research has shown that DNN models can be very brittle in the presence of input noise and variations. Our work is a first step towards improving the robustness of medical imaging models, with a particular focus on the kinds of noise encountered in medical settings. Although our experimental setup focuses on models for classifying chest radiographs, the techniques we propose are worth exploring in other medical imaging applications.

While the RoMIA framework achieves considerable improvements in robust accuracy, there still remains a gap in accuracy on clean and noisy inputs, especially for high levels of noise, that could be addressed by future work. One possible direction is to address robustness when training from scratch, in contrast to RoMIA, which only addresses it in the transfer learning step. Also, our work evaluates robustness as accuracy in classifying photographs of chest radiographs (i.e., the CheXphoto dataset). Future work could evaluate robustness under a broader set of conditions. Another interesting direction would be evaluating these techniques in a broader range of medical imaging applications. Given the criticality of medical imaging applications, robustness evaluation should be made a standard part of the regulatory evaluation process for these models. Finally, human checking of the output of AI models is one way of improving the confidence in their decisions. This could be enabled by creating explainable models that produce a human-interpretable justification for their decisions. Addressing these issues will go a long way towards enabling the adoption of AI-based medical imaging in clinical practice.

## Data Availability

The raw data supporting the conclusions of this article will be made available by the authors, without undue reservation.
